# Pesticide Flow Analysis to Assess Human Exposure in Greenhouse Flower Production in Colombia 

**DOI:** 10.3390/ijerph10041168

**Published:** 2013-03-25

**Authors:** Camilo Lesmes-Fabian, Claudia R. Binder

**Affiliations:** Department of Geography, Ludwig Maximilian University of Munich, Luisenstrasse 37 D-80333, Munich, Germany; E-Mail: claudia.binder@lmu.de

**Keywords:** dermal exposure assessment, respiratory exposure assessment, pesticides, material flow analysis, greenhouses, developing countries, Colombia, flower crops

## Abstract

Human exposure assessment tools represent a means for understanding human exposure to pesticides in agricultural activities and managing possible health risks. This paper presents a pesticide flow analysis modeling approach developed to assess human exposure to pesticide use in greenhouse flower crops in Colombia, focusing on dermal and inhalation exposure. This approach is based on the material flow analysis methodology. The transfer coefficients were obtained using the whole body dosimetry method for dermal exposure and the button personal inhalable aerosol sampler for inhalation exposure, using the tracer uranine as a pesticide surrogate. The case study was a greenhouse rose farm in the Bogota Plateau in Colombia. The approach was applied to estimate the exposure to pesticides such as mancozeb, carbendazim, propamocarb hydrochloride, fosetyl, carboxin, thiram, dimethomorph and mandipropamide. We found dermal absorption estimations close to the AOEL reference values for the pesticides carbendazim, mancozeb, thiram and mandipropamide during the study period. In addition, high values of dermal exposure were found on the forearms, hands, chest and legs of study participants, indicating weaknesses in the overlapping areas of the personal protective equipment parts. These results show how the material flow analysis methodology can be applied in the field of human exposure for early recognition of the dispersion of pesticides and support the development of measures to improve operational safety during pesticide management. Furthermore, the model makes it possible to identify the status quo of the health risk faced by workers in the study area.

## 1. Introduction

Pesticides are chemicals of growing public health concern because epidemiological studies have found that they are associated with different cancers [1,2,3,4], neurologic pathologies [5,6,7], respiratory symptoms [8] and hormonal and reproductive abnormalities [9,10,11,12,13]. Regardless of the risks involved in using pesticides, they are still considered necessary for agriculture because they allow intensive production [14]. Therefore, it is crucial to assess the risk due to pesticide use to improve their management and to reduce exposure, thereby protecting human health. 

Floriculture is a growing agricultural activity in countries such as Argentina, Colombia, Ecuador, Mexico, India, Kenya and Zimbabwe, where greenhouse environment conditions are designed to optimize plant growth [15,16]. Colombia is the world’s second largest flower exporter, with a cultivated area of 6,800 hectares and an average of 15 workers per hectare [17]. Studies in the 1990s showed birth defects among children as well as adverse reproductive outcomes in populations occupationally exposed to pesticides in the floriculture crop system in Colombia [18,19]. Although the floriculture industry has made significant progress in reducing pesticide exposure, and numerous studies have assessed exposure to pesticides in greenhouses worldwide [16,20,21,22,23,24,25,26,27,28,29,30,31,32,33], there have been no recent studies of human exposure in the floriculture system in Colombia. 

Tools for dermal exposure, such as EASE [34], EUROPOEM [35], PHED [36], RISKOFDERM [37], COSHH [38] STOFENMANAGER [39] and the approaches proposed by the U.S. EPA [40], are targeted at occupational situations in industrial processes in Europe and the USA, but they do not consider agricultural processes such as pesticide management. DREAM [41] and DERM [42] are methods focused on occupational activities in pesticide management in developing countries; nonetheless, their semi-quantitative estimations still lack reliability and validity [42,43]. Teubl [44] applied the methods PHED, RISKOFDERM, DERM and DREAM to estimating dermal exposure in the potato farming system in Colombia, and the results showed that each model delivers a different dermal exposure score because of the different determinants considered in each model, resulting in uncertainties about the real risk of exposure. Therefore, taking into account the disadvantages of the existing methodologies, a tool is required to provide a quantitative unambiguous estimation of dermal and inhalation pesticide exposure in developing countries. 

Material flow analysis (MFA) is a method to describe and analyze the material and energy balance of a firm, a region, or a nation. It is based on the law of matter conservation and is defined by a geographic system boundary, a time span within which the analysis is performed, processes which depict human activities, and flows of goods, matter, or energy between these processes [45]. It has been applied to different processes such as the balance of durables in developing countries [46], the tracing of pollutants through environmental systems such as watersheds or urban regions [47,48,49,50] and the flow of metals [51,52,53,54]. Accordingly, this methodology might be applied in the field of human exposure, allowing quick and early recognition of the fractioning of the pesticides in the human body during pesticide management activities and helping to identify activities that are crucial to improving operational safety. 

The goals of this study were the following: (i) to investigate the feasibility of the application of the material flow analysis methodology (MFA) to the field of human exposure to pesticides, (ii) to develop a tool that helps to estimate dermal and inhalation exposures to pesticides, and (iii) to identify pesticide management activities or processes that could be improved in the floriculture system in Colombia.

To achieve these goals, the following research questions were addressed:

(1) How can the material flow analysis methodology be adapted to study human exposure to pesticides in agricultural systems?
(2) What are the advantages and disadvantages of using this methodology in the field of human exposure and risk assessment of pesticide use?
(3) Based on the model outputs, what is the current situation with respect to human exposure to pesticides in the flower crop systems in Colombia, and how can the management of human exposure to pesticides be improved?

## 2. Methodology

### 2.1. Material Flow Analysis

**Figure 1:**
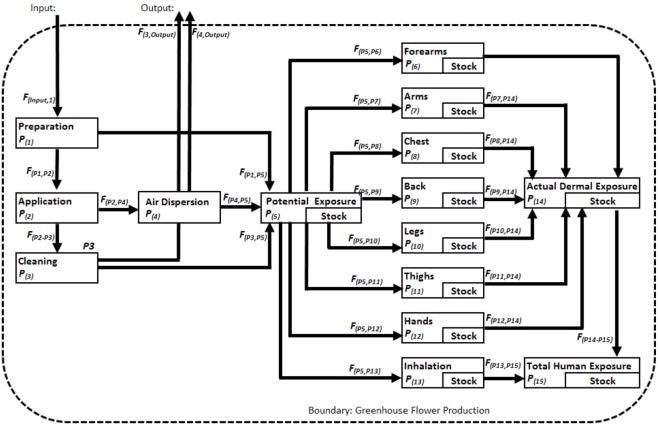
Pesticide flow analysis for the floriculture system (P: Processes, F: Flows).

The MFA method [55,56] is based on the mass conservation law and studies the flow of a substance among the different processes involved in a system. In our particular case, the method was applied to analyzing the flow of pesticides in the floriculture system during pesticide management activities such as preparation, application and cleaning of pesticide application equipment. Human exposure to pesticides was studied in terms of the fractionation of pesticides in the human body, including the dermal and inhalation exposure routes (Figure 1). The floriculture system was defined in terms of the pesticide-related activities that are performed in the greenhouse (preparation and application of the pesticides) and the cleaning rooms (where all the application and personal protection equipment is cleaned). 

This study focused only on the pesticide flow to the human body; therefore, the flow to target plants, soil and air were considered outputs of the system. The system is composed of 15 processes and 25 fluxes. The pesticide enters the system as *input* and flows according to three pesticide management activities: preparation (*P_1_*), application (*P_2_*) and cleaning (*P_3_*). These are considered transportation processes without a stock. From the preparation and cleaning, there is a direct transport of pesticide to the different body parts (*P_5_*). During the application, there is a transport of the pesticide to the air (*P_4_*) and to the different body parts (*P_5_*). The potential dermal exposure (PDE), *P_5_*, is the sum of the PDE from *P_1_*, *P_2_*, and *P_3_.* This is defined as the fraction of contaminant landing on the outer layer of the personal protective equipment [57]. The actual dermal exposure (ADE), *P_14_*, is defined as the amount of contaminant reaching exposed skin surfaces [57]. The level of protection given by the personal protective equipment is defined in the model separately for each body part in *P_6_* to *P_13_*. The pesticide flow between the potential (*P_5_*) and actual exposure (*P_14_*) depends on the level of substance retention given by the personal protective equipment. The retained amount of pesticide is defined in the model as the stock of *P_6_* to *P_13_*. The inhalation exposure (*P_13_*) is defined as the amount of contaminant arriving at the inhalation mask, and the stock is the amount retained by the filters used in the protection mask. The actual inhalation exposure is the amount of contaminant that crosses the filter in the mask. 

The pesticide flow among all the processes is defined by a mass balance and is expressed by the following equations proposed by Baccini and Brunner, 2012 [56]:

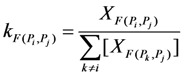
(1)

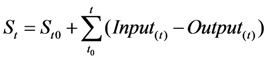
(2)


The transfer coefficient *k* for any flow from *P_i_* to *P_j_* is giving by Equation (1), where *X_F_*_(*Pi, Pj*)_ is the amount of pesticide flowing from *P_i_* to *P_j_*, Σ[*XF*_(*Pk, Pi*)_] is the sum of the amounts of pesticide flows coming to *P_i_*, *S_t_* is the stock after time step *t*, *t_0_* is the time of initial time step *t*, *t* is the current time step and *S_t0_* is the existing stock at the initial time step. The time step is defined as one working day of 8 h. The transfer coefficients were obtained by means of field measurements explained in the following sections. 

### 2.2. Description of the Study Area

**Figure 2:**
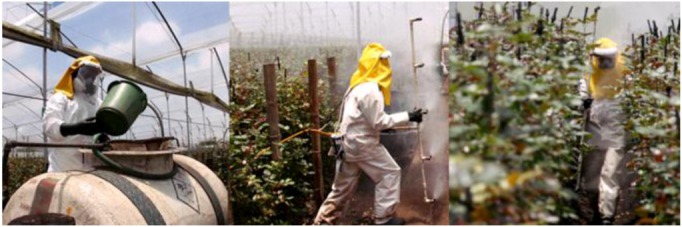
Preparation (**left**) and application of pesticide (**central** and **right**). in a greenhouse for flower production in Colombia.

The study area selected for the measurement of the pesticide flows was a farm dedicated mostly to rose production, with an area of 25.5 ha, located on the Bogota Plateau at 2,685 m.a.s.l. The average temperature is 13 °C, and inside the greenhouses, the temperature fluctuates during the day from 6 to 11 °C at 6:00 am, 21 to 31 °C at 11:00 am and 22 to 29 °C at 2:00 pm. The rose plants had a crop density of 8.2 to 8.6 plants/m^2^ in rows 32 m long and 0.8 m wide, separated by 0.6 m paths. A greenhouse has between 170 and 230 rows. The main pests affecting the rose crop production are downy mildew (*Peronospora sparsa*), grey mold (*Botrytis cinerea*), thrips and spider mites (*Tethranycus spp.*). Fungicide management is performed using a rotation of products such as carbendazim (0.6 cc/L), carboxin-thiram (1 cc/L), mancozeb (2 cc/L), dimethomorph (0.7 cc/L) propamocarb chlorohydrate (1.8 cc/L) and mandipropamide (0.8 cc/L). The pesticide preparation is made on the field mixing the commercial pesticide products with water in a 500-L container. The pesticides were applied using a standard personal protection equipment used by all the farms registered as members of the Association of Colombian Flower Exporters. It consisted of a rubber level B Hazmat suit (a garment that protects against splashes from hazardous chemicals with an external breathing mask, hood, rubber gloves and waterproof boots). The cleaning activity consists of washing the personal protective equipment and the application accessories in a washing facility by using water and cleaning products like detergent and soap. Figure 2 shows an example of pesticide management in greenhouse rose production and Table 1 lists the main characteristics of these pesticides. 

### 2.3. Data Measurement

#### 2.3.1. Dermal Exposure Measurement

The pesticide flows were measured during the three pesticide management activities: preparation, application and cleaning (*P_1_* to *P_3_*). The pesticide fractioning in the human body (*P_6_* to *P_12_*) was measured by means of the *whole body dosimetry* method [59,60,61] using the tracer uranine (fluorescein sodium salt; C_20_H_10_Na_2_O_5_; CAS Registry Number: 518-47-8; PubChem Compound ID: 10608) as a surrogate for the pesticides. The selection of this tracer was based on its low detection level, rapid quantification, solubility in spray mixtures, minimal physical effects on droplet evaporation, distinctive properties differentiating it from background or naturally occurring substances, stability, moderate cost, nontoxicity and acceptability under the regulations of the US Food and Drug Administration [62].

**Table 1:** Characteristics of the fungicides used in the case study during the study period.

Commercial Name	Active Ingredient	Chemical Group	% of Active Ingredient	Dose	Total AI Applied (g/d)	Confirmed Health Effects [58]	Possible Health Effects [58]
Bavistin	Carbendazim	Benzimidazole	50%	0.6 g/L	728	Reproduction/development effects	Endocrine disrupter
Carbovax	Carboxin	Oxathiin	20%	1 g/L	447	Eye irritant	Carcinogen, reproductive/development effects
	Thiram	Dithiocarbamate	20%	1 g/L	447	No information available	Carcinogen, mutagen, endocrine disrupter, reproduction/development effects, respiratory tract, eye and skin irritant
Dithane	Mancozeb	Dithiocarbamate	100%	2 cc/L	2400	Carcinogen, respiratory tract irritant, reproduction/development effects	Mutagen, endocrine disrupter, skin irritant
Forum	Dimethomorph	Morpholine	50%	0.7 g/L	878	Respiratory tract, eye and skin Irritant	Reproductive/development effects
Previcur	Propamocarb Hydrochloride	Carbamate	53%	1.8 g/L	2,365	Skin irritant	Acetyl cholinesterase inhibitor
	Fosetyl	Organophosphate	31%	1.8 g/L	1,383	Eye irritant, reproduction/development effects	Carcinogen, acetyl cholinesterase inhibitor, neurotoxicant
Revus	Mandipropamid	Mandelamide	25%	0.8 g/L	480	Skin irritant	No information available

**Figure 3:**
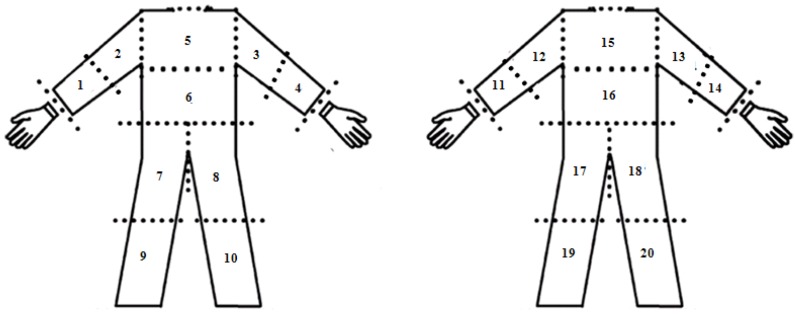
Tyvek^®^ cutting scheme (adapted from [61]).

In addition, previous studies of human exposure to pesticides have demonstrated the advantages of and positive results obtained with the tracer uranine [63,64]. Tyvek^®^ garments (DuPont™) and cotton gloves were used as sampling media. Before the test, Tyvek^®^ garments were labeled by body part (Figure 3): arms, forearms, thighs, legs (left, right, frontal and dorsal leg parts), chest, abdomen and back (upper and lower back part), and when the evaluated activities were finished, the Tyvek^®^ garments were cut according to the labeling scheme and were packed and conserved in a dark place. The same procedure was followed for the gloves. The measurement of the potential exposure was performed once a day washing the personal protective equipment in order to avoid residual contamination of uranine between the measurements. The different personal protective equipment parts were currently used by the farm whose appropriate condition is monitored by the occupational hygiene department in the farm.

The field measurements were carried out between 11:00 am and 2:00 pm. The duration of the preparation, application and cleaning activities were, as an average, 15, 8 and 30 min, respectively. In the model these times were extrapolated to 1 h. The application of pesticides was made by motorized equipment consisting of a Bean^®^ Pump (Model No. R-10; Max RPM: 580; HP: 3.4; GPM: 10.0; PSI: 500; KW: 2.5; LPM: 37). The spraying was performed with 5 nozzles (Ref: C-35) with a flow rate of 3 L/min, mounted in a pipe 1.60 m long. The nozzles were spaced 40 cm apart in the pipe (See Figure 2). Following the normal pesticide application procedure, 3 workers performed the application at the same time, each holding a pipe, spraying sidewards and walking forwards.

In the laboratory, following a previously developed protocol [63,64], the uranine in the Tyvek^®^ sections and gloves was first extracted by shaking all pieces in glass bottles with 400 mL of ultrapure water. Afterward, aliquots of 2 mL of the extraction solution, together with aliquots from the samples in the tracer solution in a 500 L container, were taken in cuvettes, and three drops of 1 mol NaOH were added. Finally, the measurement of uranine was performed using a Perkin Elmer LS 50-B Luminescence Spectrometer at an excitation wavelength of 491 nm, an emission wavelength of 520 nm, an excitation slit of 10 nm, an emission slit of 10 nm, an integration time of 1 s, and an emission filter cut-off at 515 nm. A series of standard concentrations (*i.e.*, 0.05, 0.1, 0.5, 1, 3, 5 and 10 ppb) were used for the calibration of the instrument. The detection limit of the instrument was in the range of 0.05 to 30 ppb. When concentrations were above this detection limit, dilutions were made to 50× or 2,500×.

PDE was measured on three different days during the preparation, application and cleaning processes. The PDE was calculated as the ratio of the amount of uranine measured in the Tyvek^®^ garment (*U_T.O_*) plus the amount of uranine measured in the gloves (*U_G_*), divided by the total amount of uranine applied measured in the 500-L container (*U_A_*), according to Equation (3):

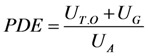
(3)
where U_T.O_ was calculated as the sum of the amounts of uranine measured on the different Tyvek^®^ pieces according to Equation (4) through Equation (6):

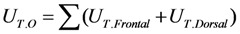
(4)

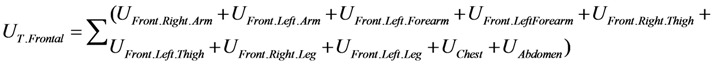
(5)

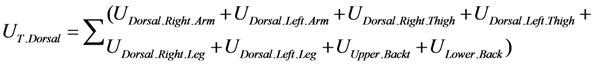
(6)


Because the application is the activity that contributes with more than 99% to the total exposure [40,63], ADE was measured only during the application with the three workers wearing the Tyvek^®^ garments under the personal protective equipment. ADE was measured on three different days during the application activity, with the participation of the same three workers performing the application simultaneously and using the respective sampling media. ADE was calculated as the ratio of the amount of uranine measured in the Tyvek^®^ garment over the total amount uranine applied measured in the 500 L container. 

The level of protection (PF: Protection Factor) for each body part was calculated as the fraction of pesticide retained by the barrier of the personal protective equipment. It was calculated only for the application activity as the ratio of the ADE to the PDE, according to Equation (7):

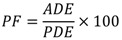
(7)


#### 2.3.2. Inhalation Exposure Measurement

The inhalation exposure was measured using the *button personal inhalable aerosol sampler* (BPIAS). It was chosen because of its efficiency and precision, according to previous studies involving evaluation of the level of occupational exposure to inhalable airborne substances [65,66,67]. The inhalation exposure measurement was performed at the same time as the dermal exposure measurement. During the application, two workers carried sets of breathing equipment consisting of one Leland Legacy^®^ Single Pump (calibrated to sample air at a rate of 15 L/min) connected to a BPIAS that contained a filter paper with a porosity of 25 µm. The filter papers were collected, labeled and packed for analysis in the laboratory. The amount of uranine measured in the filters represented the potential inhalation exposure. In addition, filters were located in the inner structure of the inhalation masks. These filters were also collected to determine the actual inhalation exposure. The protection factor given by the mask was calculated in the same way as the protection factor for dermal exposure, according to Equation (7). The measurement was performed twice during the two applications (*i.e.*, ADE and PDE) on three different days, for a total of 12 measurements. 

#### 2.3.3. Exposure Assessment in the Study Region

Based on the transfer coefficients obtained from the field measurements and the amount of pesticide applied per person during an 8-h work day over an evaluated pesticide management period of six weeks, the pesticide flow analysis model was first used to assess the risk of exposure to the fungicide mancozeb and then to assess the risk of exposure to the fungicides carbendazim, carboxin, dimethomorph, mandipropamide, propamocarb chlorhydrate, and thiram. The dermal absorption estimates were based on the actual dermal exposures calculated with the pesticide flow model and the absorption reference values for each pesticide reported in the AERU Pesticide Properties Database [58]. The estimated dermal absorption values were compared with acceptable operator exposure level (AOEL) values, which are health-based limits established on the basis of the full toxicological assessment required for pesticide registration and represent the quantity of pesticide that can be absorbed daily over a lifetime without manifesting toxic effects. These exposure level values allow quantification of the risk for pesticide operators [58].

## 3. Results

### 3.1. Pesticide Flow Analysis

**Figure 4:**
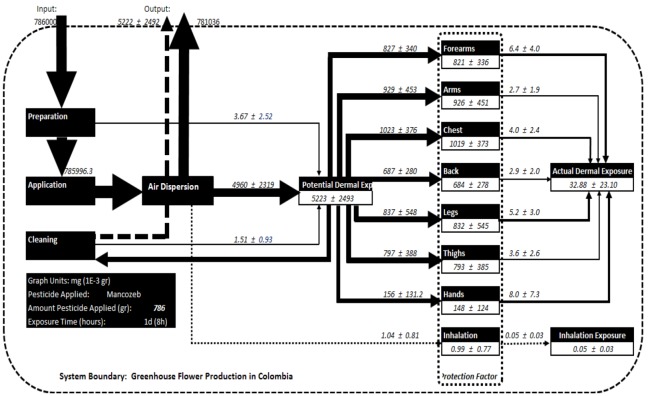
Pesticide flow analysis for the fungicide mancozeb. The units are in mg during an exposure time of 8 h. The transfer coefficients of the model are provided in the Appendix.

Figure 4 shows the pesticide flow analysis for mancozeb when 786 cc of active ingredient were applied (the average of 25 applications for the evaluated pesticide management period of six weeks) during a work day of 8 h. 

The model shows that the exposure was very high during the application step, contributing 99.9% to the total PDE, while the preparation step contributed 0.07% and the cleaning step contributed 0.03. The exposure during preparation and cleaning is due to accidental splashes that cause minimal exposure compared with the application activity, in which most of the pesticide solution is used and during which the exposure is very high. Nevertheless, despite the high PDE (5,223 ± 2,493 mg/d), the ADE was very low (32 ± 23 mg/d), which indicates a level of protection of approximately 95% for the hands and between 99.2 and 99.8% for the rest of the body parts. 

With respect to ADE, the model shows that the forearms and hands were the most exposed body parts (*i.e.*, 8.0 ± 7.3 and 6.4 ± 4.0, respectively). This shows that despite the high level of protection given by the personal protective equipment, there is a leak of pesticide solution droplets through the overlap between gloves and sleeves. This same situation occurs for the legs, whose ADE values (5.2 ± 3.0 mg/d) might be due to a leak of pesticide solution droplets through the overlap between boots and trousers, and for the chest, whose ADE values (4.0 ± 2.4 mg/d) might be due to a leak of pesticide solution droplets through the buttons. 

### 3.2. Health Risk in the Study Area

Table 2 shows the daily average dermal absorption estimates for the eight pesticides evaluated (*i.e.*, carbendazim, carboxin, mancozeb, dimethomorph, propamocarb, mandipropamide, thiram and fosetyl). The dermal absorption of mancozeb was estimated at 3.6 ± 2.5 mg/d. This was based on the ADE results (32 ± 23 mg/d) and the dermal absorption value of 11% for mancozeb [58]). This value is greater than the AOEL reference value of 2.45 mg/d, which suggests that there is a health risk faced by the operator. Similar findings were found for carbendazim, thiram and mandipropamide. The inhalation exposure was found to be 0.05 ± 0.03 mg/d, which compared with the AEOL reference value, can be considered negligible and does not represent a health risk. 

**Table 2:** Estimated actual dermal and inhalation exposures for 8 evaluated pesticides used in greenhouse flower crops in Colombia.

Commercial Name	Active Ingredient (AI)	* Average Applied/ Operator (cc/d)	Actual Dermal Exposure (mg/d)	Inhalation Exposure	Dermal Absorption (%) [58]	Estimated Pesticide Absorbed (mg/d)	AOEL (mg/d)
Bavistin	Carbendazim	485	20.2 ± 14.2	0.03 ± 0.02	10	2.0 ± 1.4	1.4
Carbovax	Carboxin	716	29,2 ± 21.0	0.05 ± 0.03	5	1.5 ± 2.1	3.85
	Thiram	745	31.1 ± 21.9	0.05 ± 0.03	10	3.1 ± 2.1	1.4
Dithane	Mancozeb	786	32.8 ± 23.1	0.05 ± 0.03	11	3.6 ± 2.5	2.45
Forum	Dimethomorph	585	24.4 ± 17.2	0.04 ± 0.03	20	4.8 ± 3.4	10.5
Previcur	Propamocarb	1,480	61.9 ± 43.5	0.09 ± 0.06	10	6.1 ± 4.3	-
	Fosetyl	1,488	61.9 ± 43.5	0.09 ± 0.06	1	0.6 ± 0.4	350
Revus	Mandipropamide	640	26.7 ± 18.8	0.04 ± 0.03	10	2.6 ± 1.8	2.45

**Figure 5:**
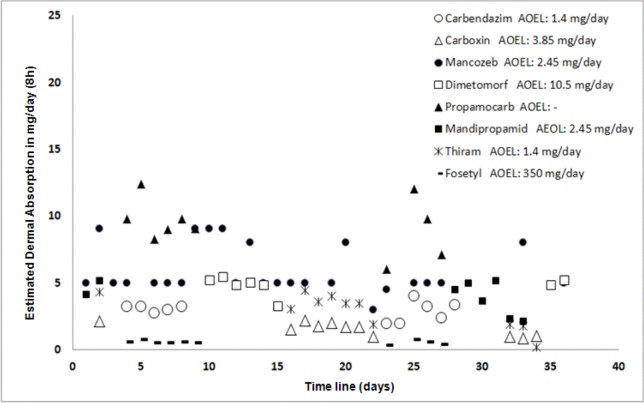
Estimated daily dermal absorption of pesticides for the evaluated pesticide management period of six weeks. Estimations are based on the actual dermal exposures (arithmetic mean, n = 9) calculated with the pesticide flow model and the absorption reference values for each pesticide reported in the AERU Pesticide Properties Database [58].

* This average of the amount of active ingredient applied was obtained for the evaluated pesticide management period of six weeks (Figure 5): carbendazim, n = 10; carboxin, n = 11; thiram, n = 11; mancozeb, n = 25; dimethomorph, n = 9; propamocarb, n = 10; fosetyl, n = 10; mandipropamide, n = 8.

## 4. Discussion

### 4.1. Pesticide Flow Analysis Approach

This paper presented a pesticide flow analysis modeling approach based on the material flow analysis methodology. The pesticide flow model helps to identify the patterns of pesticide distribution on the body, the level of protection given by personal protective equipment and estimates of potential and actual dermal and inhalation exposure to pesticides. This information can be used to determine the health risk level by comparing the model estimates with the AEOL reference values for each pesticide. In addition, the model makes it possible to easily identify the activities or body parts that have high levels of exposure, which is useful in identifying improvements that will decrease exposure during pesticide management. However, the model outcomes correspond to a certain interval of time and do not consider issues such as pesticide accumulation or pesticide degradation rate. Furthermore, the model considers each pesticide separately and does not take into account the facts that pesticides are usually applied in mixtures and that this might alter the chemical nature of the pesticides. 

### 4.2. Pesticide Management in the Case Study

One characteristic of the greenhouse flower crop system in Colombia is pesticide application with five nozzles mounted on a 1.60 m long pipe. Previous studies [29] have shown that the distribution of the PDE on the body parts depends on the spray direction of the nozzle (Table 3), and because the application in the study area was made sideways with five nozzles simultaneously, body parts were exposed homogenously, with the exception of the hands. This fact is reflected in the results of the PDE distributions, which range between 13 and 19% for the body parts and 3% for the hands. These results are different from those obtained in previous studies in which only one nozzle was used and the application was made downward, forward or backward, and the exposures differ, with high values generally found on the lower body parts [29]. 

**Table 3:** Comparison of the distribution of PDE for different application techniques. The values represent the percentages of the PDE distributions on the body parts. Technique 1 corresponds to the present study and techniques 2–4 correspond to experiments made in greenhouse pepper crops in Spain and Greece [29].

Body Parts	PDE (% in Body)			
	1. Spray Sideways with 5 Nozzles	2. Spray Gun Downward	3. Spray Lance Forward	4. Spray Lance Backward
Back	13.1	0.5	0.8	1.4
Chest	19.5	0.8	1.5	1.9
Arm	17.7	18.8	10.0	6.0
Forearm	15.7	13.3	7.3	10.0
Thighs	15.2	12.6	11.3	8.1
Legs	15.9	46.7	55.1	27.0
Hands	3.0	7.3	14.0	45.6
Total	100.0	100.0	100.0	100.0

Concerning the ADE distribution, previous studies have shown similar results in which the hands and forearms are the most exposed body parts, and dermal exposure is the main contributor of the total exposure [68,69].

Another characteristic of this study was that the study area was the size of the paths between the crop rows, which is only 60 cm wide, creating a close space in which the sprayed pesticide droplets move (Figure 2). This issue might contribute to the homogenous potential dermal exposure. This contrasts with the paths of greenhouse production systems in other locations [29], which are between 1 and 1.5 m wide. 

### 4.3. Health Risk in the Study Area

Daily dermal absorption estimations were higher than AEOL reference values for mancozeb, carbendazim, thiram and mandipropamide. Taking into account that environmental conditions like humidity affect the level of absorption [69], the health risk might be higher for these pesticides during long periods of time. Figure 5 shows that during the six-week pesticide management period evaluated, carbendazim and thiram were applied 11 times, mancozeb was applied 25 times and mandipropamide was applied eight times. 

Because of this application frequency and the possibility of being exposed to a group of pesticides with different toxicity levels, the health risk might be higher. Furthermore, in the flower production system, additional pesticides with different toxicity levels are applied, which suggests that there might be an even greater potential health risk. For instance, in a previous survey of 84 greenhouse flower farms in Colombia, 14.3% of the pesticides were found to belong to category I, 14.4% to category II, 52% to category III and 19.2% to category IV [70]. This suggests that the health risk assessment might be different depending on the toxicity level of each pesticide and the application frequency.

## 5. Conclusions

The material flow analysis methodology can be applied in the field of human exposure for estimation of the patterns of pesticide distribution on the human body during different pesticide management activities. This methodology not only assesses the level of exposure but also provides information on potential measures for improving operational safety during pesticide management. Furthermore, the model outcomes, together with pesticide information such as AOEL reference values, can be used to assess the health risk associated with pesticide exposure. 

Our pesticide flow model integrates three activities and two routes of exposure during pesticide management, which is different from other approaches in which a model was developed separately for each process or activity. Although the model can be applied to case studies in regions with similar characteristics, such as the application technique, the infrastructure and the type of personal protection equipment, the model should be calibrated when these characteristics change. Although the model provides static information about the exposure during one 8-h work day, further improvements are necessary to improve the health risk assessment by including in the model time-dependent issues such as the cumulative exposure over several days and the pesticide degradation rate.

With respect to the status quo of health risk in the case study, of the eight pesticides evaluated, mancozeb, carbendazim, thiram and mandipropamide were found to represent a health risk to operators because their dermal absorption estimates exceeded the AOEL reference values. However, this health risk might be reduced by using adequate personal protective equipment and improving the protection in overlapping areas such as between gloves and sleeves and between boots and trousers. There might also be a significant health risk reduction achieved by using pesticides with lower toxicity levels and by reducing the application frequency of the same pesticides, especially if their toxicity levels are very high. 

## Appendix

**Table A1:** Transfer coefficients used for the pesticide flow analysis model according to the field measurements of the tracer uranine.

	PDE	ADE			Stock		
**Body Parts**							
Forearms (n = 9)	1.84E−05 ± 7.57E−06	1.43E−07 ± 8.83E−08			1.83E−05 ± 7.48E−06		
Arms (n = 9)	2.07E−05 ± 1.01E−05	6.10E−08 ± 4.19E−08			2.06E−05 ± 1.00E−05		
Chest & Abdomen (n = 9)	2.28E−05 ± 8.37E−06	8.94E−08 ± 5.30E−08			2.27E−05 ± 8.32E−06		
Back (n = 9)	1.53E−05 ± 6.24E−06	6.47E−08 ± 4.37E−08			1.52E−05 ± 6.20E−06		
Thighs (n = 9)	1.77E−05 ± 8.63E−06	7.95E−08 ± 5.81E−08			1.77E−05 ± 8.57E−06		
Legs (n = 9)	1.86E−05 ± 1.22E−05	1.16E−07 ± 6.72E−08			1.85E−05 ± 1.21E−05		
Hands (n = 9)	3.48E−06 ± 2.92E−06	1.79E−07 ± 1.62E−07			3.30E−06 ± 2.76E−06		
Total Dermal (n = 9)	1.17E−04 ± 5.60E−05	7.32E−07 ± 5.14E−07			1.16E−04 ± 5.55E−05		
Inhalation (n = 12)	2.31E−08 ± 1.80E−08	1.10E−09 ± 8.50E−10			2.20E−08 ± 1.72E−08		
**Pesticide Management Activities**							
Preparation (n = 3)	4.67E−06 ± 3.21E−06						
Application (n = 9)	1.10E−04 ± 5.16E−05						
Cleaning (n = 3)	1.92E−06 ± 1.18E−06						

## References

[1] De Roos A.J., Zahm S.H., Cantor K.P., Weisenburger D.D., Holmes F.F., Burmeister L.F., Blair A. (2003). Integrative assessment of multiple pesticides as risk factors for non-Hodgkin’s lymphoma among men. Occup. Environ. Med..

[2] Hardell L., Eriksson M., Nordström M. (2002). Exposure to pesticides as risk factor for Non-Hodgkin’s lymphoma and hairy cell leukemia: Pooled analysis of two Swedish case-control studies. Leuk. Lymphoma.

[3] Infante-Rivard C., Sinnett D. (1999). Preconceptional paternal exposure to pesticides and increased risk of childhood leukaemia. Lancet.

[4] Richter E.D., Chlamtac N. (2002). Ames, pesticides, and cancer revisited. Int. J. Occup. Environ. Health.

[5] Baldi I., Cantagrel A., Lebailly P., Tison F., Dubroca B., Chrysostome V., Dartigues J.F., Brochard P. (2003). Association between Parkinson’s disease and exposure to pesticides in southwestern France. Neuroepidemiology.

[6] Baldi I., Lebailly P., Mohammed-Brahim B., Letenneur L., Dartigues J.F., Brochard P. (2003). Neurodegenerative diseases and exposure to pesticides in the elderly. Am. J. Epidemiol..

[7] Elbaz A., Levecque C., Clavel J., Vidal J.S., Richard F., Amouyel P., Alpérovitch A., Chartier-Harlin M.C., Tzourio C. (2004). CYP2D6 polymorphism, pesticide exposure, and Parkinson’s Disease. Ann. Neurol..

[8] Salameh P.R., Baldi I., Brochard P., Raherison C., Abi Saleh B., Salamon R. (2003). Respiratory symptoms in children and exposure to pesticides. Eur. Respir. J..

[9] Weidner I.S., Møller H., Jensen T.K., Skakkebæk N.E. (1998). Cryptorchidism and hypospadias in sons of gardeners and farmers. Environ. Health Perspect..

[10] Bell E.M., Hertz-Picciotto I., Beaumont J.J. (2001). Case-cohort analysis of agricultural pesticide applications near maternal residence and selected causes of fetal death. Am. J. Epidemiol..

[11] Garry V.F., Harkins M.E., Erickson L.L., Long-Simpson L.K., Holland S.E., Burroughs B.L. (2002). Birth defects, season of conception, and sex of children born to pesticide applicators living in the Red River Valley of Minnesota, USA. Environ. Health Perspect..

[12] Garry V.F., Holland S.E., Erickson L.L., Burroughs B.L. (2003). Male reproductive hormones and thyroid function in pesticide applicators in the Red River Valley of Minnesota. J. Toxicol. Environ. Health Part A.

[13] Hanke W., Jurewicz J. (2004). The risk of adverse reproductive and developmental disorders due to occupational pesticide exposure: An overview of current epidemiological evidence. Int. J. Occup. Med. Environ. Health.

[14] Glass C.R., Machera K. (2009). Evaluating the risks of occupational pesticide exposure. Hell. Plant Prot. J..

[15] Illing H.P.A. (1997). Is working in greenhouses healthy? Evidence concerning the toxic risks that might affect greenhouse workers. Occup. Med..

[16] Ribeiro M.G., Colasso C.G., Monteiro P.P., Filho W.R.P., Yonamine M. (2012). Occupational safety and health practices among flower greenhouses workers from Alto Tietê region (Brazil). Sci. Total Environ..

[17] (2010). ASOCOFLORES, Colombian Florriculture Report. Colombian Association of Flower Exporters.

[18] Restrepo M., Munoz N., Day N.E., Parra J.E., de Romero L., Nguyen-Dinh X. (1990). Prevalence of adverse reproductive outcomes in a population occupationally exposed to pesticides in Colombia. Scand. J. Work Environ. Health.

[19] Restrepo M., Muñoz N., Day N., Hernandez C., Blettner M., Giraldo A. (1990). Birth defects among children born to a population occupationally exposed to pesticides in Colombia. Scand. J. Work Environ. Health.

[20] Cerrillo I., Olea-Serrano M.F., Ibarluzea J., Exposito J., Torne P., Laguna J., Pedraza V., Olea N. (2006). Environmental and lifestyle factors for organochlorine exposure among women living in Southern Spain. Chemosphere.

[21] Costa C., Silva S., Coelho P., Roma-Torres J., Teixeira J.P., Mayan O. (2007). Micronucleus analysis in a Portuguese population exposed to pesticides: Preliminary survey. Int. J. Hyg. Environ. Health.

[22] Herńndez A.F., Mackness B., Rodrigo L., López O., Pla A., Gil F., Durrington P.N., Pena G., Parrón T., Serrano J.L., Mackness M.I. (2003). Paraoxonase activity and genetic polymorphisms in greenhouse workers with long term pesticide exposure. Hum. Exp. Toxicol..

[23] Jurewicz J., Hanke W., Sobala W., Ligocka D. (2008). Dermal exposure to pesticides among women working in Polish greenhouses using cotton patches (*Ekspozycja dermalna na pestycydy kobiet pracujacych w gospodarstwach ogrodniczych - Wyniki badań z wykorzystaniem próbników bawełnianych*). Med Pr..

[24] Machera K., Goumenou M., Kapetanakis E., Kalamarakis A., Glass C.R. (2003). Determination of potential dermal and inhalation operator exposure to malathion in greenhouses with the whole body dosimetry method. Ann. Occup. Hyg..

[25] Monsó E., Magarolas R., Badorrey I., Radon K., Nowak D., Morera J. (2002). Occupational asthma in greenhouse flower and ornamental plant growers. Am. J. Respir. Crit. Care Med..

[26] Rosano A., Gemelli V., Giovannelli C., Paciotti G., Sabatucci A., Spagnolo A. (2009). Fertility changes in women working in greenhouses (*Alterazione della fertilità nelle lavoratrici in serra*). Med Lav..

[27] Gerth van Wijk R., Patiwael J.A., de Jong N.W., de Groot H., Burdorf A. (2011). Occupational rhinitis in bell pepper greenhouse workers: Determinants of leaving work and the effects of subsequent allergen avoidance on health-related quality of life. Allergy Eur. J. Allergy Clin. Immunol..

[28] Flores A.P., Berenstein G.A., Hughes E.A., Zalts A., Montserrat J.M. (2011). Pesticide risk assessment in flower greenhouses in Argentina: The importance of manipulating concentrated products. J. Hazard. Mater..

[29] Nuyttens D., Braekman P., Windey S., Sonck B. (2009). Potential dermal pesticide exposure affected by greenhouse spray application technique. Pest Manag. Sci..

[30] Ramos L.M., Querejeta G.A., Flores A.P., Hughes E.A., Zalts A., Montserrat J.M. (2010). Potential Dermal Exposure in greenhouses for manual sprayers: Analysis of the mix/load, application and re-entry stages. Sci. Total Environ..

[31] Lu J.L. (2005). Risk factors to pesticide exposure and associated health symptoms among cut-flower farmers. Int. J. Environ. Health Res..

[32] Munnia A., Puntoni R., Merlo F., Parodi S., Peluso M. (1999). Exposure to agrochemicals and DNA adducts in Western Liguria, Italy. Environ. Mol. Mutagen..

[33] Esechie J.O., Ibitayo O.O. (2011). Pesticide use and related health problems among greenhouse workers in Batinah Coastal Region of Oman. J. Forensic Leg. Med..

[34] Cherrie J.W., Tickner J., Friar J. (2003). Evaluation and Further Development of the EASE Model 2.0.

[35] van Hemmen J.J. (2001). EUROPOEM, a predictive occupational exposure database for registration purposes of pesticides. Appl. Occup. Environ. Hyg..

[36] Dosemeci M., Alavanja M.C.R., Rowland A.S., Mage D., Hoar Zahm S., Rothman N., Lubin J.H., Hoppin J.A., Sandler D.P., Blair A. (2002). A quantitative approach for estimating exposure to pesticides in the agricultural health study. Ann. Occup. Hyg..

[37] van Hemmen J.J., Auffarth J., Evans P.G., Rajan-Sithamparanadarajah B., Marquart H., Oppl R. (2003). RISKOFDERM: Risk assessment of occupational dermal exposure to chemicals. An introduction to a series of papers on the development of a toolkit. Ann. Occup. Hyg..

[38] Garrod A.N.I., Rajan-Sithamparanadarajah R. (2003). Developing COSHH essentials: Dermal exposure, personal protective equipment and first aid. Ann. Occup. Hyg..

[39] Marquart H., Heussen H., Le Feber M., Noy D., Tielemans E., Schinkel J., West J., van der Schaaf D. (2008). ‘Stoffenmanager’, a web-based control banding tool using an exposure process model. Ann. Occup. Hyg..

[40] U.S. EPA (2007). Dermal Exposure Assessment: A Summary of EPA Approaches; EPA/600/R-07/040F.

[41] van-Wendel-De-Joode B., Brouwer D.H., Vermeulen R., van Hemmen J.J., Heederik D., Kromhout H. (2003). DREAM: A method for semi-quantitative dermal exposure assessment. Ann. Occup. Hyg..

[42] Blanco L.E., Aragón A., Lundberg I., Wesseling C., Nise G. (2008). The Determinants of Dermal Exposure Ranking Method (DERM): A pesticide exposure assessment approach for developing countries. Ann. Occup. Hyg..

[43] Kromhout H., van Wendel de Joode B., van Hemmen J. (2008). The accuracy of DERM may be a self-fulfilling DREAM. Ann. Occup. Hyg..

[44] Teubl S.K., Lesmes-Fabian C., Binder C.R. (2012). Evaluation of Models for Dermal Exposure Assessment in Farming Systems in Developing Countries.

[45] Binder C.R., Anderson R. (2012). Material flow analysis. Berkshire Encyclopedia of Sustainability.

[46] Binder C., Bader H.P., Scheidegger R., Baccini P. (2001). Dynamic models for managing durables using a stratified approach: The case of Tunja, Colombia. Ecol. Econ..

[47] Binder C., Schertenleib R., Diaz J., Bader H.P., Baccini P. (1997). Regional water balance as a tool for water management in developing countries. Int. J. Water Resour. Dev..

[48] Bergbäck B., Anderberg S., Lohm U. (1994). Accumulated environmental impact: The case of cadmium in Sweden. Sci. Total Environ..

[49] van der Voet E., van Egmond L., Kleijn R., Huppes G. (1994). Cadmium in the European Community: A policy-oriented analysis. Waste Manag. Res..

[50] Kleijn R., van der Voet E., Udo de Haes H.A. (1994). Controlling substance flows: The case of chlorine. Environ. Manag..

[51] Spatari S., Bertram M., Fuse K., Graedel T.E., Shelov E. (2003). The contemporary European zinc cycle: 1-year stocks and flows. Resour. Conserv. Recycl..

[52] Frosch R.A., Clark W.C., Crawford J., Sagar A., Tschang F.T., Webber A., Wilson W.R. (1997). The industrial ecology of metals: A reconnaissance [and discussion]. Philos. Trans. Math. Phys. Eng. Sci..

[53] Graedel T.E., Bertram M., Fuse K., Gordon R.B., Lifset R., Rechberger H., Spatari S. (2002). The contemporary European copper cycle: The characterization of technological copper cycles. Ecol. Econ..

[54] Gordon R.B., Graedel T.E., Bertram M., Fuse K., Lifset R., Rechberger H., Spatari S. (2003). The characterization of technological zinc cycles. Resour. Conser. Recycl..

[55] Brunner P., Rechberger H. (2004). Practical Handbook of Material Flow Analysis.

[56] Baccini P., Brunner P.H. (2012). Metabolism of the Anthroposphere—Analysis, Evaluation and Design.

[57] Rajan-Sithamparanadarajah R., Roff M., Delgado P., Eriksson K., Fransman W., Gijsbers J.H.J., Hughson G., Mäkinen M., van Hemmen J.J. (2004). Patterns of dermal exposure to hazardous substances in European Union Workplaces. Ann. Occup. Hyg..

[58] AERU (2011). Pesticide Properties Database. http://sitem.herts.ac.uk/aeru/footprint/en/index.htm.

[59] WHO (1982). Field Surveys of Exposure to Pesticides. Standard Protocol VBC/82.1.

[60] Chester G. (1993). Evaluation of agricultural worker exposure to, and absorption of, pesticides. Ann. Occup. Hyg..

[61] Hughes E.A., Zalts A., Ojeda J.J., Flores A.P., Glass R.C., Montserrat J.M. (2006). Analytical method for assessing potential dermal exposure to captan, using whole body dosimetry, in small vegetable production units in Argentina. Pest Manag. Sci..

[62] Akesson N.B., Yates W.E. (1964). Problems relating to application of agricultural chemicals and resulting drift residues. Ann. Rev. Entomol..

[63] Lesmes-Fabian C., Garcia-Santos G., Leuenberger F., Nuyttens D., Binder C.R. (2012). Dermal exposure assessment of pesticide use: The case of sprayers in potato farms in the colombian highlands. Sci. Total Environ..

[64] García-Santos G., Scheiben D., Binder C.R. (2011). The weight method: A new screening method for estimating pesticide deposition from knapsack sprayers in developing countries. Chemosphere.

[65] Chen M.R., Tsai P.J., Wang Y.F. (2008). Assessing inhalatory and dermal exposures and their resultant health-risks for workers exposed to polycyclic aromatic hydrocarbons (PAHs) contained in oil mists in a fastener manufacturing industry. Environ. Int..

[66] De Schampheleire M., Spanoghe P., Brusselman E., Sonck S. (2007). Risk assessment of pesticide spray drift damage in Belgium. Crop Prot..

[67] Witschger O., Grinshpun S.A., Fauvel S., Basso G. (2004). Performance of personal inhalable aerosol samplers in very slowly moving air when facing the aerosol source. Ann. Occup. Hyg..

[68] Vitali M., Protano C., Monte A., Ensabella F., Guidotti M. (2009). Operative modalities and exposure to pesticides during open field treatments among a group of agricultural subcontractors. Arch. Environ. Contam. Toxicol..

[69] Aprea C., Centi L., Santini S., Lunghini L., Banchi B., Sciarra G. (2005). Exposure to Omethoate during stapling of ornamental plants in intensive cultivation tunnels: Influence of environmental conditions on absorption of the pesticide. Arch. Environ. Contam. Toxicol..

[70] Varona M., Tolosa J., Cardenas O., Torres C., Pardo D., Carrasquilla G., Frumkin H. (2005). Descripcion del uso y manejo de plaguicidas en las empresas de flores afiliadas a asocoflores. Biomedica.

